# Randomised controlled pilot study to investigate the effectiveness of thoracic epidural and paravertebral blockade in reducing chronic post-thoracotomy pain: TOPIC feasibility study protocol

**DOI:** 10.1136/bmjopen-2016-012735

**Published:** 2016-12-01

**Authors:** Joyce Yeung, Teresa Melody, Amy Kerr, Babu Naidu, Lee Middleton, Kostas Tryposkiadis, Jane Daniels, Fang Gao

**Affiliations:** 1 Institute of Inflammation and Ageing, College of Medical and Dental Sciences, University of Birmingham, Birmingham, UK; 2 Academic Department of Anaesthesia, Critical Care, Pain and Resuscitation, Heart of England NHS Foundation Trust, Birmingham, UK; 3 Department of Thoracic Surgery, Heart of England NHS Foundation Trust, Birmingham, UK; 4 Birmingham Clinical Trials Unit, University of Birmingham, Birmingham, UK

**Keywords:** Perioperative Medicine

## Abstract

**Introduction:**

Open chest surgery (thoracotomy) is considered the most painful of surgical procedures. Forceful wound retraction, costochondral dislocation, posterior costovertebral ligament disruption, intercostal nerve trauma and wound movement during respiration combine to produce an acute, severe postoperative pain insult and persistent chronic pain many months after surgery is common. Three recent systematic reviews conclude that unilateral continuous paravertebral blockade (PVB) provides analgesia at least equivalent to thoracic epidural blockade (TEB) in the postoperative period, has a lower failure rate, and symptom relief that lasted months. Crucially, PVB may reduce the development of subsequent chronic pain by intercostal nerve protection or decreased nociceptive input. The overall aim is to determine in patients who undergo thoracotomy whether perioperative PVB results in reducing chronic post-thoracotomy pain (CPTP) compared with TEB. This pilot study will evaluate feasibility of a substantive trial.

**Methods and analysis:**

TOPIC is a randomised controlled trial comparing the effectiveness of TEB and PVB in reducing CPTP. This is a pilot study to evaluate feasibility of a substantive trial and study processes in 2 adult thoracic centres, Heart of England NHS Foundation Trust (HEFT) and University Hospital of South Manchester NHS Foundation Trust (UHSM). The primary objective is to establish the number of patients randomised as a proportion of those eligible. Secondary objectives include evaluation of study processes. Analyses of feasibility and patient-reported outcomes will primarily take the form of simple descriptive statistics and where appropriate, point estimates of effects sizes and associated 95% CIs.

**Ethics and dissemination:**

The study has obtained ethical approval from NHS Research Ethics Committee (REC number 14/EM/1280). Dissemination plan includes: informing patients and health professionals; engaging multidisciplinary professionals to support a proposal of a definitive trial and submission for a full HTA application dependent on the success of the study.

**Trial registration number:**

ISRCTN45041624; Pre-results.

**Strengths and limitations of this study**

- Chronic pain post-thoracotomy is common and can result in significant economic and healthcare burden. Very little is known about whether anaesthetic and analgesic technique will prevent chronic pain.
- This randomised controlled pilot study will assess patient recruitment to a definitive study.
- Results from this study will contribute towards limited evidence towards prevention of development of chronic post-thoracotomy pain.
- This pilot study will not answer the research question but will lead to well-designed definitive study.
- To maintain patient safety and clinical care, postoperative clinical teams looking after patients are not blinded to anaesthetic technique patient has received. The low risk of patients knowing their treatment allocation can potentially introduce bias. To limit bias, the outcome assessors are blinded to anaesthetic techniques and patients were not informed of treatment allocation.

## Introduction

### 

Open chest surgery (thoracotomy) is considered the most painful of surgical procedures.^1^ Forceful wound retraction, costochondral dislocation, posterior costovetebral ligament disruption, direct intercostal nerve trauma and wound movement during respiration combine to produce an acute, severe postoperative pain insult and persistent chronic pain many months after surgery is common.^1–5^ Chronic post-thoracotomy pain (CPTP) is defined by the International Association for the Study of Pain, as pain that recurs or persists along a thoracotomy incision at least 2 months following the surgical procedure.^6^ The aetiology of CPTP seems to be nociceptive and neuropathic in nature. Risk factors include female gender, younger age, psychological vulnerability and intercostal nerve damage.^7^ ^8^ CPTP can be very disabling and results in a substantial economic and healthcare burden. About 8500 surgical lung resections are performed annually in the UK mainly for lung cancer.^9^ Our literature review suggests that CPTP occurs in 43% of patients, who had no pre-existing pain problem, at 6 months after surgery. Other surveys indicate 66% of patients suffered from pain that impaired normal daily activity for at least 12 months after thoracotomy, 90% of affected patients required prescription medications for pain and anxiety while 30% received specialist treatments.^10^ About 29% of patients with CPTP have neuropathic pain that is harder to treat than somatic pain. Of these, 43% experienced some level of disruption in their employment status, including reduced working time, unemployment or early retirement.^11^ ^12^

### Current practice

Thoracic epidural blockade (TEB) is currently regarded as the ‘gold standard’ for pain relief in thoracotomy; however, this dogma has recently been challenged. Recent evidence from two meta-analyses and systematic reviews comparing the analgesic efficacy and side effects of epidural versus paravertebral blockade (PVB) for thoracotomy pain control concluded that although the analgesia was comparable, PVB had a better short-term side effect profile, including urinary retention, hypotension, nausea and vomiting and pulmonary complications.^13^ ^14^

Despite the evidence, previous surveys of clinical practice have consistently demonstrated that thoracic epidural remained the most popular choice. A survey of Australian thoracic anaesthetists in 1997 revealed that 79% regarded TEB as the method of choice for analgesia in thoracotomy.^15^ Similar results were found in the UK with 80% of anaesthetists considered TEB as the best mode of pain relief for upper abdominal surgery.^16^ A 2011 survey of 39 thoracic units in the UK that was carried out by the Association of Cardiothoracic Anaesthetists (ACTA) reported that the majority of thoracic anaesthetists (2/3 units) prefer TEB to PVB, which suggests that most thoracic anaesthetists have yet to be convinced by the evidence available.^17^

### Effect of anaesthesia and analgesic technique

The physiological response to surgically induced tissue injury is analogous to an acute systemic inflammatory response. This is pertinent to thoracotomy, during which musculoskeletal disruption from retraction, intercostal nerve injury and pleural breach is impossible to avoid even with meticulous surgical technique. It is almost certain that the interaction of these factors results in the high prevalence of CPTP.^8^ ^18^ ^19^ The somatic afferent neuronal traffic generated by surgery is integrated at spinal cord level before onward transmission to the higher central nervous system. Elaboration of this input via the thalamus and onward to the cerebral cortex results in the sensation of localised acute pain and the psychological and emotional responses of distress. This afferent information can be modulated by therapeutic nerve blockade or a reduction in its humoral consequences, for example, by the addition of anti-inflammatory agents. Nerve block reduces acute symptoms by preventing pain transmission. It may also reduce the complex ‘elaboration’ of pain pathways at a spinal cord level and thus desensitise pathways that underpin the development of chronic pain. Preventing this sensitisation is proposed as the basis for the so-called ‘Pre-emptive analgesia’.^11^ If spinal cord sensitisation does play a role in CPTP, it follows that the less excitatory information transmitted to spinal cord level, the greater the chance of chronic pain prevention. Although TEB and PVB use local anaesthetics to reduce afferent input, their sites of action are different. TEB is a central neuraxial blockade, effective at spinal cord level bilaterally. It does not induce complete neural ‘Silence’ but reduces onward transmission by a combination of local anaesthetic-induced sodium channel blockade and opioid interaction in the substantial gelatinosa. In contrast, the effect of PVB is dependent on local anaesthetic-mediated prevention of peripheral nociceptive afferent traffic reaching the spinal cord.^20^ ^21^ In this sense, quiescence of this neuronal input may be more complete with an effective PVB. There is therefore a sound theoretical basis to hypothesise divergent effects of the two techniques on cord sensitisation and subsequent CPTP generation.

### The evidence for the comparative effectiveness of PVB and TEB

Three recent systematic reviews conclude that unilateral continuous PVB provides analgesia at least equivalent to TEB in the postoperative period, has a lower failure rate, and symptom relief that lasted months.^13^ ^14^ PVB resulted in fewer pulmonary complications, less urinary retention, hypotension and nausea/vomiting.^22^ In 2005, in a multicentre UK audit of 365 pneumonectomies, PVB was associated with significantly lower major postoperative complications (23% vs 35%) and lower unexpected intensive care unit admissions (8% vs 18%) compared with TEB.^23^ The benefits seen with PVB can be explained by the blocking of unilateral intercostals nerves only, with preservation of respiratory and sympathetic function on the contralateral side. These reviews were updated in October 2012 with six additional trials, five of which^20^ ^22–25^ (total n=244) supported the conclusions of the systematic reviews; however, a small trial found the median morphine consumption significantly higher with PVB (n=12) than the TEB group (n=12) (9 vs 36 mg, p=0.003).^20^ Crucially, PVB may reduce the development of subsequent chronic pain by intercostal nerve protection or decreased nociceptive input.^21^

Previous trials directly comparing TEB and PVB have not examined chronic pain as the primary outcome and as a result, evidence that PVB is superior in preventing CPTP is derived from other sources. PVB has long been used as a treatment (rather than prevention) of CPTP to good effect, with symptom relief lasting months. Observational studies have reported lower chronic pain rates after PVB relative to TEB, albeit with non-randomised methodology. Local anaesthetic-induced PVB has been proven to abolish cortical somatosensory-evoked potentials from thoracic dermatome stimulation.^21^ There is no evidence for an equivalent abolition in TEB. Prevention of afferent input to the central nervous system is known to be important in pain modulation. Total blockade of somatosensory-evoked potentials by PVB removes the stimulus for central sensitisation and could be uniquely effective in preventing CPTP from being triggered. There are many parallels between CPTP and chronic pain after breast surgery with recent trial evidence suggesting that PVB exerts a beneficial effect in chronic pain prevention.^20^ ^26^

The most recent Cochrane Review comparing PVB and TEB in adults undergoing thoracotomy found no difference between PVB and TEB in 30-day mortality following surgery.^27^ PVB was associated with a lower incidence of pneumonia and delirium when compared with TEB. No significant difference between PVB and TEB was found in critical care admission and there were insufficient data to compare the two techniques in terms of cardiovascular complications or the need for further surgery. In terms of analgesic efficacy, PVB was comparable to TEB and was found to be superior at 24 hours postoperatively. PVB also had a better minor complication profile with lower incidence of hypotension, nausea and vomiting, pruritus and urinary retention. No difference between PVB and TEB was found in excessive sedation and length of hospital stay. There were insufficient data to compare PVB and TEB in terms of assessing CPTP and health costs.

The review also concluded that a well-conducted randomised controlled trial (RCT) comparing PVB and TEB in thoracotomy is needed. Areas that require further research include 30-day mortality, major complications, chronic pain and health costs.

### Study rationale

CPTP is unpleasant and disabling. Surveys have indicated 66% of patients suffered pain that impaired their normal daily activity for at least 12 months after thoracotomy.^10^ Ninety per cent of affected patients required prescription medications for pain and anxiety. Of these, 43% experienced disruption in their employment status. CPTP certainly results in substantial economic and healthcare burden. It is expected that the number of patients suffering CPTP will increase following the rise in number of lung resections over the last decade (around 60%) in the UK and Ireland. There is now an urgent need to answer this important research question for benefits to patients and the NHS.

If one technique proves to be significantly better, our results will influence national policy and directly improve patient care. Our results will also be applicable to the prevention of chronic postsurgical pain from other one side operations, such as hernia repair, leg amputation, gallbladder removal or breast surgery.

### Study aim

The overall aim of this research is to determine in adult patients who undergo open chest operation whether perioperative PVB at thoracotomy results in reducing CPTP compared with TEB. To answer this research question with authoritative evidence of clinical and cost-effectiveness of PVB, a multicentre RCT with a parallel health economic evaluation is required.

However, feasibility studies are the best way to assess feasibility of a large, expensive full-scale study, and in fact are an almost essential prerequisite. Conducting feasibility prior to the main study can enhance the likelihood of success of the main study and potentially help to avoid doomed main studies.^28^ We have therefore designed this multicentre feasibility study comparing the effectiveness of TEB and PVB in reducing CPTP. This study will evaluate feasibility of a substantive trial and study processes by making the following qualitative and quantitative assessments.

### Objectives for the feasibility study

The aims of the feasibility stage are to assess various aspects of the trial design and management and not to determine the relative effectiveness of PVB and TEB.

#### Primary objective

To establish the number of patients randomised as a proportion of those eligible to enter the study.

#### Secondary objectives

1. Assessment of effectiveness of patient identification and screening processes
2. Identification and analysis of any reasons for failure to recruit patients
3. Examination of the educational materials provided to surgeons and anaesthetists to ensure they are fit for purpose
4. Assessment of willingness of surgeons and anaesthetists to participate
5. Assessment of the effectiveness of the randomisation process of patients
6. Assessment of sustainability of single blinding of patients to treatment allocation
7. Evaluation of robustness of data collection processes during patient's hospital stay
8. The proportion of patients followed up at 6 months
9. Acceptability to and impact on patients of the interventions
10. Assessment of trial processes, including the choice of outcome measures and impact on staff
11. Derivation of the preliminary data from clinical outcome measures to inform the sample size calculation for the substantive study

## Trial design

### Design

TOPIC is an RCT comparing the effectiveness of TEB and PVB in reducing CPTP. This is a pilot study to evaluate feasibility of a substantive trial and study processes.

### Setting

The study started in July 2015 with final follow-up to end December 2016. Two adult thoracic centres, Heart of England NHS Foundation Trust (HEFT) and University Hospital of South Manchester NHS Foundation Trust (UHSM), with a patient case mix and size typical of UK thoracic anaesthetic practice, will take part in this feasibility. Based on National Thoracic Surgery Activity and Outcome Report and local audit data, an estimated total of 500 elective open thoracotomies were performed at BHH (n=400) and at UHSM (n=100) in 2011. All adult patients admitted for elective thoracotomy who fulfil the inclusion and exclusion criteria during the study period will be approached at both sites. The coordinating centre will be based within MIDRU in Birmingham Heartlands Hospital.

### Flow of participants during the trial

**Figure 1:**
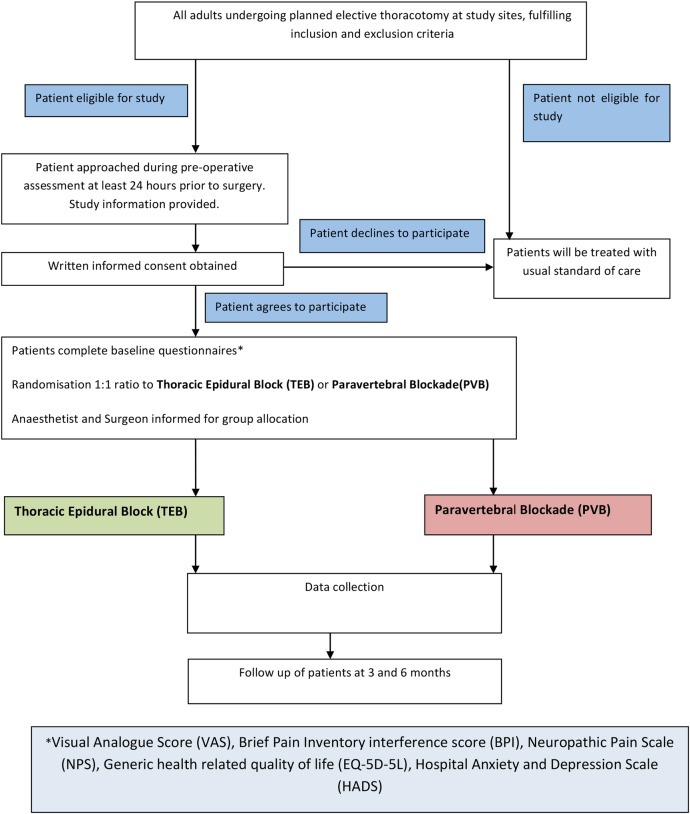
Flow of participants during the trial.

The anticipated journey of participants through the trial is depicted in the flow chart as indicated in figure 1.

All adults undergoing planned elective thoracotomy at study sites fulfilling inclusion and exclusion criteria will be approached and the trial written information sheets will be given to them and the study will be discussed fully. Written informed consent will be obtained. Patients who consent to participate in the trial will be randomised to either receiving the TEB or PVB arm which will be delivered during the patient's surgery by either a surgeon or an anaesthetist trained in the study protocol. Patients will be randomised on the morning of the surgery. If either the surgeon or anaesthetist is not available to deliver the intervention, randomisation will not go ahead.

Presurgery and postsurgery study data collection will be performed and study questionnaires will be completed, as detailed. Adverse events will be collected throughout the duration of patients' participation in the study. Table 1 is a summary of investigations and assessment.

**Table 1:** Summary of investigations and assessments

	Baseline clinic appointment prior to surgery	In-hospital				Follow-up		
		Intraoperative	Day one*	Day two*	Day three*	Hospital discharge	Three months	Six months
Eligibility and written informed consent†	X							
Demographic data	X							
Previous Medical History	X							
Randomisation	X Day of surgery							
TEB/PVB insertion data		X						
Other intraoperative data		X						
Post operative observations			X	X	X	X		
Post-operative pulmonary complications			X	X	X	X		
Visual Analogue Scale score	X		X	X	X	X	X	X
Brief Pain Inventory	X		X	X	X	X	X	X
Post-operative analgesic use			X	X	X	X	X	X
Acute Complications			X	X	X	X		
Hospital Length of Stay						X		
Mortality						If applicable		
Neuropathic Pain Scale	X					X	X	X
Discharge data and histology data						X		
EQ-5D-5L	X					X	X	X
Hospital Anxiety Depression Scale	X					X	X	X
Patient satisfaction						X	X	X
Adverse Events	If applicable							
Protocol deviations	If applicable							

*Day 1 is the first full calendar (from 12 midnight) postsurgery, day 2 is the second full calendar day, day 3 is the third full calendar day.

### Study eligibility

#### Inclusion criteria

- Aged ≥18 years
- Elective open thoracotomy
- Able to understand the study information and provide written informed consent
- American Society of Anaesthesiologists physical status I, II or III
- Not known to be pregnant

#### Exclusion criteria

- Known allergy to local anaesthetics
- Infection near the proposed puncture site
- Coagulation disorders
- Thoracic spine disorders
- Chest wall resection
- Emergency thoracic surgery
- Previous thoracotomy
- Likely inability to comply with completion of the study questionnaires

#### Patient identification and screening procedure

Research staff will work in close liaison with the multidisciplinary team responsible for routine patient care. Patients listed for elective open thoracotomy will be identified and screened for eligibility at clinics prior to their planned surgery. If a patient is screened but is not eligible for the TOPIC trial or consent for randomisation is not given, a record of the case will be kept in the screening log. The log will collect hospital number, patient's initials, date of birth, age, ethnic group, BMI and reason not eligible for the trial. The log should be kept in each study centre's site file and a copy (in an anonymised format—removing initials and hospital number) sent to TOPIC trial office. This will inform recruitment targets. No further information will be collected on ineligible patients or those that have not given consent for randomisation.

#### Patient recruitment

Ideally, consent should be sought under unhurried circumstances when entry criteria are fulfilled. Consent is sought in several stages. We aim to identify patients who will need a planned surgical thoracic operation within the two recruiting study sites. Eligible patients will be identified in clinics prior to surgery. Ethically approved participant information sheet will be given to eligible patients, supported by face-to-face discussion with the research team and their consultant. The participant information sheet has been developed with feedback from our PPI representatives, and any ambiguities, or questions frequently asked by those approached, will be collated. This will enable a comprehensive, but clear, participant information sheet to be deployed if we proceed onto a substantive trial.

If patient consents to participate in the study, written informed consent will be obtained by a member of the research team. Enough time will be given to discuss the study, ask any questions before seeking consent. If the patient decides to enter the trial, they will be asked to sign two original copies of the patient consent form which will then be countersigned by the member of the research team taking the consent. The patient will retain one copy of the signed consent form. The second copy will be photocopied and the photocopy placed in the patient's medical records while the original will be retained in the Investigator Site File.

Participants will be asked to consent to their GP being informed about their participation in the study.

#### Randomisation

After written informed consent, the patient will be randomised, on the day of surgery, to either TEB or PVB. Participants will be individually randomised into the study in an equal 1:1 ratio. Randomisation will be by a web-based randomisation system, with a telephone option available as back-up, managed by the Birmingham Clinical Trials Unit (BCTU).

A ‘minimisation’ procedure using a computer-based algorithm and incorporating a random element will be used to avoid chance imbalances in the following variables. The variables chosen are:

- gender,
- age <65 or ≥65 years,
- centre (Birmingham Heartlands Hospital or University Hospital of South Manchester),
- thoracotomy for lung cancer resection or for other indication.

Using the web-based randomisation service, patients will be allocated to a treatment group. The anaesthetist and surgeon in charge of patient care will be informed of the patient's allocation. A unique study identification number will be assigned to the participant.

#### Study anaesthetic and analgesic strategies

All study patients will be anaesthetised by experienced thoracic anaesthetists (consultants) who have been trained and deemed competent in both anaesthetic techniques. The study team has worked closely with consultant anaesthetists to develop a suitable training package. Consultant anaesthetists are capable to perform epidurals and PVBs; however, for the purpose of the study, anaesthetists will be asked to perform the techniques to the standard required by study protocol. Two online training videos detailing TEB and PVB have been produced alongside supplementary written step-by-step guide. A copy of the videos is also available in DVD format. All anaesthetists participating in the study must review video and/or written material and confirm that they are able to perform the techniques according to study protocol. Further training, if required, will be provided by study-designated trainers at each participating sites who can demonstrate and observe performance if required. All training material will be freely available at each site and will act as a reference for participating anaesthetists and surgeons. Training by participating anaesthetists will be documented in training logs.

To be pragmatic, some variation in technical aspects of block insertion detailed in the training is anticipated, between experienced thoracic anaesthetists, and those trained for the trial, and between centres, as anaesthetists will use their judgement on the best techniques for each patient. This represents real-world variation in anaesthetic practices and will not contribute to bias since randomisation will ensure balance across groups by centre. The location and dose of anaesthetic will be captured on a postoperative case report form (CRF).

#### Experimental group: PVB

Three single injections, awake or asleep, using a 16 G/18 G graduated epidural needle with 15 mL 0.25% bupivacaine at T3–4, 5–6 and 7–8, will be given preoperatively. The PVB catheter will be placed at T5 under direct vision by a surgeon at the end of surgery before chest closure. A loading dose of 10 mL 0.25% bupivacaine is given before chest closure followed by infusion of 0.125% bupivacaine 0.1–0.25 mL/kg/hour. See online supplementary appendix for further details.

10.1136/bmjopen-2016-012735.supp1 Supplementary appendix

#### Control group: TEB

Usual practice of TEB, awake or asleep, using a 16 G/18 G graduated epidural needle with a catheter inserted at the spinal level supplying the skin at the incision site, a test dose of 3 mL of 0.25% bupivacaine, and a loading dose of 0.25% bupivacaine 0.1 mL/kg with up to 3 mg of diamorphine. This will be followed by infusion of 0.125% bupivacaine with 2 μg/mL fentanyl at 0.1–0.25 mL/kg/hour. See online supplementary appendix for further details.

#### Study treatment dispensing

All anaesthetics and analgesia will be taken from standard theatre pharmacy stock. As TOPIC does not fall under the Medicines for Human Use (Clinical Trials) regulations 2004, segregated stocks for trial use and specific trial labelling are not required. Temperature monitoring should follow local pharmacy practice and deviations need not be reported to the TOPIC Study Co-ordinator.

#### Blinding of trial allocations

By the nature of the interventions, it is not possible to conceal treatment assignments from surgeons and anaesthetists. Moreover, from a safety aspect, it is vital that the nursing staff caring for the patient know the amount of epidural opiates prescribed before administering systemic opiates, and known adverse events such as hypotension or pruritus expected to arise from the respective anaesthetic approaches.

Every attempt will be made to blind study participants to their group allocation. The epidural or PVB infusion catheter will be taped laterally on the side of operation, so no visible difference can be seen by the patient. Infusion pumps used by both groups will also be identical.

#### Withdrawal from the trial

Withdrawal from the trial before surgery is a decision of the participant; however, withdrawn patients can bias trial results and reduce the power of the trial to detect important differences, so randomisation will take place as close to the time of surgery as is practical in order to reduce postrandomisation withdrawals. Following surgery, participants should be encouraged to allow clinical data collection to continue even if they decline to complete further questionnaires.

Cessation of the allocated anaesthetic strategy will also be necessitated in cases where a known serious adverse reaction to the anaesthetic occurs or a suspected unexpected serious adverse reaction occurs.

#### Protocol violations

Any incidences of study participants not receiving the anaesthetic strategy allocation by randomisation will be recorded. All study and protocol violations and deviations will be documented in the patients CRF and reported to the Study Sponsor via the Trial Office. Patients will be analysed according to group allocation, by intent-to-treat analysis.

#### Additional intraoperative analgesia

Supplementary intraoperative analgesia will not be restricted and can follow local policy. Analgesia and doses will be recorded as part of the study in the patients CRF.

#### Postoperative analgesia

Both groups should continue with TEB/PVB infusion of 0.1–0.25 mL/kg/hour bupivacaine, in the first instance for 48 hours postoperatively. All participants will receive regular paracetamol and prophylactic antiemetics unless contraindicated. Non-steroidal anti-inflammatory drugs can also be administered if appropriate. All analgesic requirements will be recorded during inpatient follow-up.

For the TEB group, intravenous morphine boluses will be prescribed for breakthrough pain which is not relieved by the epidural top ups. If the epidural is ineffective and no block is evident, the TEB can be reinserted at the discretion of the anaesthetic team. If pain relief is inadequate, morphine PCA (patient-controlled analgesia) can be administered.

For the PVB group, intravenous morphine boluses followed by morphine PCA will start on recovery from anaesthesia.

## Outcomes and data collection

### Patient recruitment into study

The overall aims of the feasibility are to find out if a larger trial is feasible. The quantitative measurements related to this include

- proportion of all elective thoracic procedures screened,
- proportion of eligible participants of those screened,
- proportion of eligible participants randomised.

In this feasibility study of two centres, HEFT and University of south Manchester NHS Foundation Trust, there would be an approximate total of 500 elective open thoracotomies over the study period. The plan will be to recruit and randomise as many patients as possible over the 12-month study period. It is expected that between 50 and 75 eligible patients will be recruited from 2 sites.

### Patient identification and screening

We would expect a very high proportion of patients to be screened across both study sites, given that only patients with planned thoracotomy will be included. The proportion of patients screened for eligibility and recorded on a screening log will be assessed and reported as proportion of patients screened from the total number of planned thoracotomies during the study period.

### Reasons for failure to recruit

The proportion of patients that were missed, which should be minimal, and the proportion of patients who decline to take part will be recorded. Patients decline for many reasons, which should be captured whenever possible. We will consent declining patients to a short interview. The reasons for declining will be recorded anonymously and analysed by the research team. If there is a strong patient preference, the substantive trial may not be feasible, similarly if this population is disinterested or conversely, taking part in other trials that preclude concurrent participation.

### Educational materials and training of surgeons and anaesthetists

Feedback on the appropriateness, value and acceptability of the training will be elicited from the feasibility sites, to enable refinement of the training programme for the substantive study, and to define a minimum competence. The training material will be evaluated for its ease of use should it be used in the substantive study.

### Evaluation of willingness of anaesthetists and surgeons to participate

As part of preparation of the study site, all anaesthetists and surgeons in both sites will be approached to evaluate willingness to participate in the trial. The Site PI(s) and the trial coordinator will discuss the protocol to ensure that all inclusion/exclusion criteria and technical aspects are well understood by the participating anaesthetists and surgeons. Patient ‘vignettes’, typical and unusual, will be presented during this training to establish whether uncertainty exists and therefore randomisation is ethical in all situations, or whether there are somewhere either technique is preferred. Training material will be revised, as per the feedback for use in the substantive study, portraying best practice in approaching and consenting participants.

The study team will also conduct a repeat national survey to assess willingness from the clinical community nationally towards the end of feasibility study.

### Effectiveness of randomisation process

This would be ascertained by the speed in which patients can be randomised and whether important prognostic data can be collected preoperatively.

### Assessment of data collection process

Assessment and identification will be made for loss of data during inhospital stay to improve data collection process for the substantive trial.

### Assessment of sustainability of single blinding of patients to treatment allocation

By the nature of the interventions, it is not possible to conceal treatment assignments from surgeons and anaesthetists. Every attempt will be made to blind study participants to their group allocation and various methods may be considered. The patient-reported outcomes will be collected remote in time from the acute intervention. There is no reason to suspect that recipients of the randomised intervention have strong preconceptions with regard to the relative effectiveness of each analgesic technique. In this feasibility study, patients will be asked at 3 and 6 months after surgery via questionnaire which technique they think they received to test if our various methods for patient blinding were effective.

### Assessment of follow-up rates

The primary outcome of the substantive study is chronic pain assessed at 6-month postrandomisation. It is therefore vital for the appropriate measures to be in place to minimise the loss of follow-up.

The research team will demonstrate and assist the patient to complete the questionnaires in person when the baseline data are collected. This face-to-face assistance and support in filling the questionnaire will help encourage patients and increase their confidence in completing questionnaires after discharge.

The patient has consented to be contacted by post or by telephone for follow-up purposes. Prior to the follow-up questionnaires being sent to patients at home, their vital status will be confirmed by a research team member from study sites. The contact information and patient status will be faxed from study sites to BCTU for follow-up purposes. Follow-up questionnaire will include pain questionnaires, patient satisfaction questionnaire and assessment of single blinding. To be viable as a primary outcome, we would expect to achieve a response rate of 80% of expected patients, using various methods of contact. We should be able to capture 100% of mortality data via NHS tracing services. A withdrawal from follow-up of over 10% would be disappointing. The reasons for loss of follow-up if any will be documented and reported at the end of the feasibility study.

### Patient-reported outcomes

At baseline and prior to surgery, five sets of questionnaires will be completed. These comprise: Visual Analogue Scale score, Brief Pain Inventory interference score (BPI),^29^ ^30^ Neuropathic Pain Scale (NPS),^31^ Generic health-related quality of life (EQ-5D-5L)^32^ and Hospital Anxiety and Depression Scale (HADS).^33^

Inhospital data collection will include Visual Analogue Scale scores, Brief Pain Inventory, analgesic use, any acute complications conducted on day 1, day 2 and day 3 postsurgery. Using day of surgery as day 0, day 1 is defined as the first full calendar day (from 12 midnight) postsurgery, day 2 is the second full calendar day and day 3 is the third full calendar day.

On hospital discharge, take home analgesia (TTOs), inhospital mortality, acute complications, unplanned admission to level 2 or level 3 care, including organ support and length of level 2/level 3 stay and total length of hospital stay. Assessment and identification will be made for loss of data during inhospital stay to improve data collection process for the substantive trial.

Six sets of questionnaires will be completed prior on hospital discharge and at 3 and 6 months postrandomisation. The national registry will be checked to confirm patients status prior to follow-up questionnaires being sent at 3 and 6 months. These questionnaires are Patient satisfaction questionnaire with their overall care and with their pain relief and question to assess whether patient was aware of treatment allocation, Visual Analogue scale scores, BPI,^29^ ^30^ NPS,^31^ Generic health-related quality of life (EQ-5D-5L)^32^ and HADS.^33^

### Acceptability to and impact on patients

Patient interviews will explore the acceptability of the intervention to patients and any impacts on their stay in hospital and postdischarge. Semistructured qualitative interviews will be undertaken with up to 30 study patients with representation of patients taking part across the 2 sites. The interviews will be conducted at 6–8 weeks postdischarge. This will allow for a reasonable recovery period postsurgery and will enable interviews to be undertaken with the small proportion of patients who go on to need chemotherapy, prior to this treatment beginning. The interviews will be performed by telephone in order to minimise the disruption to and effort required by patients.

All patients will be eligible for interview and will be selected using maximum variety sampling by age, sex and ethnic group.^34^ The need for a maximum variety sample will be balanced against spacing the interviews as evenly as possible across the 12 months of the trial so that any variations in how the trial is implemented are reflected in the patient sample. Interviews will be conducted until saturation is achieved, which is likely to be around 30 patients.^35^

A framework for the patient interviews will be developed in months 1–3 of the trial set-up period, with reference to the literature on similar trials. The framework will also be discussed with Clinical Research Ambassador Group (CRAG) based within HEFT. It will include five core questions that will be asked of all patients, which will cover:

- Reasons for taking part in the trial
- Assessing whether patients knew which anaesthetic strategy they received
- The effectiveness of staff and written communication about the trial
- How the trial impacted on their stay in hospital and at home following discharge
- Suggestions for making improvements to the recruitment processes

The semistructured nature of the interviews will allow patients to raise issues which may not have been anticipated by the research team, and will allow the interviewer to explore any patient concerns in depth. The interviews are expected to last an average of 15–20 min, and will be recorded digitally. If during the interviews, any patients indicate that they have unresolved concerns or clinical symptoms, they will be directed to their named research nurse. Similarly, if patients get upset, the interviewer will ask for the patient's consent to be contacted by their dedicated research nurse for further discussion.

Telephone interviews will also be undertaken with up to 10 patients who declined to take part in the trial, to explore their reasons for declining and to identify how a larger trial could be adapted to encourage higher rates of participation.

### Assessment of trial processes and impact on staff

Semistructured qualitative interviews with clinical and research staff will be undertaken to explore the effectiveness and efficiency of the trial processes. This will include exploring a number of the secondary outcomes:

- The effectiveness of the patient identification and screening processes
- Identification of reasons for failure to recruit patients
- The willingness of surgeons and anaesthetists to take part
- The effectiveness of the randomisation process

Interviews will also ask for staff ideas for improvement in trial processes, and explore whether there are any unintended consequences of the trial procedure which might have an impact on patient care processes or the organisation and management of care.

Up to 20 staff interviews will be undertaken, which will be spread evenly across the two sites and will include the main clinical and managerial roles affected by the trail. The interviews will be undertaken in the month following the discharge of the last trial patient home. The interviews are expected to last an average of 20–30 min, and will be recorded digitally Data Collection and Management.

All data for an individual patient will be collected by each Principal Investigator or their delegated nominees and recorded in the study-specific data collection forms (CRF). Participants will only be identified through their unique trial number allocated at the time of randomisation and their initials. Data will be collected from the time the patient is entered into the trial through their discharge from hospital and up to 6 months postsurgery.

## Statistics and data analysis

### Sample size calculation

We expect to recruit between 50 and 75 patients depending on the number we find eligible for the study. For example, we estimate that there will be ∼500 open elective thoracotomies over 12 months from the 2 sites (HEFT and UHSM), of which 60% will be eligible (300). Using our own target criteria of 25% recruited would make 75 participants. This number will allow us to measure the recruitment rate with 95% CI of width ∼10%. It will also be enough to estimate the SD of VAS score with 95% CI of width 7 points (assuming the SD is around 25 points).

### Data analysis

The size of this study will not allow reliable assessment of the effect of the intervention on outcomes and so hypothesis testing is not proposed. Analyses of feasibility and patient-reported outcomes will primarily take the form of simple descriptive statistics (eg, proportions and IQRs, means and SDs) and where appropriate, point estimates of effects sizes (eg, mean differences and relative risks) and associated 95% CIs.

In the first instance, for patient-reported outcomes, participants will be kept in the groups they were allocated, regardless of compliance with treatment (intention-to-treat). Analysis will be completed once all patients have completed 6-month follow-up. A Statistical Analysis Plan will be generated for review by the Trial Oversight Committee before any analysis takes place.

### Handling missing data

There is a potential for some missing data to occur at follow-up; however, a member of the research team will contact patients for any missing data (eg, questionnaire) via telephone and post. Where patients attend for follow-up clinic, the potential for missing data will again be limited, and the secondary outcome data will also be collected at this point. Imputation of missing responses is not proposed for patient-reported outcome as this is not a definitive trial and no hypothesis testing will be performed

### Data management and quality assurance

#### Data management and confidentiality

Personal data and sensitive information required for the TOPIC feasibility study will be collected directly from trial participants and hospital notes on data collection forms, coded with the participant's unique trial number and initials. All other patient identifiable information will be removed. Participants will be asked for their consent to transfer this information, including their name and contact address for follow-up to the BCTU office based in University of Birmingham. The data collected will be entered onto a secure computer database by BCTU staff. This database, once completed, will be locked under the direction of LM (Senior Statistician) for analysis.

All personal information received in paper format for the trial will be held securely and treated as strictly confidential according to NHS policies. All staff involved in the study (clinical, academic, BCTU) share the same duty of care to prevent unauthorised disclosure of personal information. No data that could be used to identify an individual will be published. Data will be stored on a secure server at BCTU under the provisions of the Data Protection Act and/or applicable laws and regulations. The trial coordinator, study statistician and the data manager will have access to the database until completion of the analysis. Data may be accessed by external regulatory agencies and the Study Sponsor representatives and permission for this access will be documented within the participants consent form.

#### Data quality assurance and validation

The study will adopt a centralised approach to monitoring data quality and compliance. A computer database will be constructed specifically for the study data and will include range and logic checks to prevent erroneous data entry. Independent checking of data entry of paper questionnaires will be periodically undertaken on small subsamples. The trial statistician (LM) will regularly check the balance of allocations by the stratification variables. Source data verification will only be employed if there is reason to believe data quality has been compromised, and then only in a subset of practices.

Quality assurance will begin with a clearly documented staff training programme. A register of staff who have been trained, and their competence assessed will be maintained, and only staff whose names appear on this list will be permitted to undertake study procedures. Staff will also receive regular update training and periodic reassessment of their competence. Real-time reports will be available to staff indicating missing test and questionnaire data for all participants at that centre. This will be supplemented by regular reminders from the TOPIC Trial Office for incomplete data.

#### Monitoring and audit

The study will be monitored and/or audited by HEFT under their remit as Sponsor and other regulatory bodies to ensure adherence to Good Clinical Practice and the NHS Research Governance Framework for Health and Social Care (2nd edition).

Monitoring of study data shall include confirmation of informed consent; source data verification; data storage and data transfer procedures; local quality control checks and procedures, back-up and disaster recovery of any local databases and validation of data manipulation. The trial coordinator, or where required, a nominated designee of the Sponsor, shall carry out monitoring of study data as an ongoing activity.

The first study participant who has been randomised, received surgery and completed up to the 72 hour follow-up stage of the protocol will be monitored by the Sponsors QA Manager to ensure the protocol is fit for purpose and review protocol adherence. Monitoring of study participants by the Sponsors QA manager will then occur at random intervals throughout the study based on recruitment.

Study conduct will be subject to systems audit of the Study Record for inclusion of essential documents; permissions to conduct the trial; Study Delegation Log; CVs of study staff and training received; local document control procedures; consent procedures and recruitment logs; adherence to procedures defined in the protocol (eg, inclusion/exclusion criteria, timeliness of visits); accountability of study materials and equipment calibration logs. This will be led by the trial coordinator and reported back to the Sponsor and the Sponsorship Oversight Committee.

Entries on CRFs will be verified by inspection against the source data. A sample of CRFs (10%) will be checked on a regular basis for verification of all entries made. In addition, the subsequent capture of the data on the study database will be checked. Where corrections are required, these will carry a full audit trail and justification.

Study data and evidence of monitoring and systems audits will be made available for inspection by the regulatory authority as required.

#### Long-term storage of data

Trial data will be stored archived after the formal closure of the trial in accordance with archive policy and for the appropriate duration as per current legislation.

The computer database may be stored within the BCTU and will be processed according to their trial archiving policies.

## Sponsorship and indemnity

HEFT will act as the Sponsor to this study. Delegated responsibilities will be assigned to the Chief Investigator and the NHS Trust(s) taking part in this study. The non-commercial model clinical trials agreement will be used with all participating sites detailing their local responsibilities.

HEFT holds standard NHS Hospital indemnity and insurance cover with NHS Litigation Authority for NHS Trusts in England, which apply to this study.

## Regulatory approvals

The study has obtained ethical approval from NHS Research Ethics Committee (REC number 14/EM/1280).

## Study dissemination

This feasibility study is designed to identify if a substantive trial is possible. Although a definitive answer to the key research question on effectiveness of PVB on CPTP cannot be provided, the findings of this feasibility study will be of scientific interest to others in their own right. The feasibility study will be registered on clinical trials database (http://www.clinicaltrials.gov). We plan the dissemination strategy in three aspects. The first will ensure that patients and health professionals are informed of the feasibility findings; the second will engage multidisciplinary professionals to support a proposal of a definitive RCT and the third will be to resubmit for a full HTA application dependant on the success of the feasibility study.
