# Supplementary material for: Randomised controlled pilot study to investigate the effectiveness of thoracic epidural and paravertebral blockade in reducing chronic post-thoracotomy pain: TOPIC feasibility study protocol
Source: BMJ Open. 2016 Dec 1;6(12):e012735. doi: 10.1136/bmjopen-2016-012735 (PMC5168654; doi:10.1136/bmjopen-2016-012735)
Supplement: Supplementary appendix [file bmjopen-2016-012735supp_appendix.pdf]

APPENDIX

Thoracic Epidural Blockade Template

General points on Insertion of TEB Catheter

- Institute full monitoring according to AAGBI guidelines.
- TEB can be inserted in patients awake or asleep, sitting or in lateral position
- Catheter insertion should be at mid thoracic level (T6-T7 or T7-T8)

Intra operative Utilisation of TEB catheter

- First dose is given with 3-5 ml of 0.25% bupivacaine with 2-3 mgs of diamorphine. 2mg for patients <50kg, 2.5mg for patients 50-65kg, 3mg for patients >65kg. Dose of diamorphine should be titrated if patient is more than 75 years of age.
- This mixture provides adequate analgesia for the initial skin incision and further boluses of local anaesthetics are only given if patient's physiological parameters warrants.
- Towards the end of the operation, we start our epidural infusion of 0.125% bupivacaine and 4mcg/ml fentanyl at a rate 0.1-0.25 ml/kg/h.
- All patients receive intravenous Paracetamol and NSAIDs if there are no contraindications.

Post operative Utilisation of TEB catheter

- The patient is assessed in recovery and if they have pain, further titrated boluses of 3-5mls of epidural mixture (0.125% bupivacaine with 4mcg/ml fentanyl) is given for break through pain. Bolus can be repeated.
- All thoracotomy patients are looked after in a thoracic surgical HDU. The acute pain team reviews the patients regularly and the epidural is stepped down to oral/IV analgesics after 48 hours.
- Patients are prescribed regular oral analgesics such as paracetamol and NSAIDS.
- Nursing staff regularly assesses the block height and epidural rate is titrated as per the local pain protocol.
- If the blood pressure is persistently low and other surgical causes of low blood pressure have been ruled out, the diagnosis of epidural associated hypotension is made. Metaraminol infusion is then started at 0.5-1.5 micrograms/kg body wt min^{-1} (Appendix). This avoids the need for CVC line perioperatively and restricts the amount of fluid administered.

- The pain scores at rest and when mobile, motor block, postoperative nausea and vomiting and sedation scores are also assessed regularly and recorded.
- During the post operative period any complications of epidural analgesia are noted by surgical nursing staff. Advice from the acute pain team and the anaesthetist should be sought if pain control is problematic.
- In the event when epidural is deemed ineffective, morphine boluses including morphine PCA should be prescribed.

Paravertebral Blockade Template

General points on PVB and catheter insertion

- Institute full monitoring according to AAGBI guidelines.

- PVB can be performed on patients awake or asleep, sitting or in lateral position (we prefer lateral position, asleep)

- 3 preoperative PVB injection using landmark technique at the level of T4-5, T7-8, T9-10 followed by surgical catheter insertion after the thoracotomy

Intra operative Utilisation of PVB

- 15 ml 0.25% bupivacaine with or without adrenaline (1:200000-400000) to be used for each preincisional block using landmark technique ("predetermined distance technique")

- This concentration and volume should provide adequate spread and analgesia for the initial skin incision on an patient under light general anaesthesia, who is otherwise able to tolerate one lung anaesthesia.

- We do not assume that the surgical analgesia provided by the local injections lasts longer than 2-4 hours, therefore the surgical paravertebral/epipleural catheter insertion should be performed after the thoracotomy in order to make continuous infusion possible. Within 2 hours, 10 ml 0.25% bupivacaine bolus to be administered via the catheter followed by 0.25% bupivacaine infusion with 10 ml/hour until the end of the operation.

- All patients receive intravenous Paracetamol and/or NSAIDs if there are no contraindications. The paravertebral group should have 1mg/ml morphine PCA infusion with 5 minutes lockout time for rescue pain-relief.

Post operative Utilisation of TEB catheter

- The patient is assessed in recovery and if they have pain the rate of the infusion can be changed in order to provide adequate pain-relief (0-15 ml/hour, depending on the patients' bodyweight: max. 2 mg/kg/4hour bupivacaine dose). In case of the need of higher dose 5 ml bolus should be administered first.

- All thoracotomy patients are looked after in a thoracic surgical HDU. The acute pain team reviews the patients regularly and the epipleural/paravertebral is stepped down to oral/IV analgesics after 48 hours.

- Patients are prescribed regular oral analgesics such as paracetamol and/or NSAIDS; iv morphine PCA should be available for rescue pain-relief (see above).

- Nursing staff regularly assess the pain score, neurological status, physiological parameters and the area of the anaesthetized chest wall. If the anaesthetized area unnecessarily large the infusion rate should be decreased by 2 ml/hours. The lowest rate should not be lower than 5 ml/hours. If the pain-relief is inadequate 5 ml bolus 0.25% bupivacaine should be administered and the rate should be increased back to the last adequate rate and continue with this rate till the catheter removal.

- If the blood pressure is persistently low or there any other sign of epidural spread or local anaesthetic toxicity the infusion to be stopped immediately and the patient should be managed according to the guidelines. These events will exclude the particular patient from the study.

- The pain scores at rest and when mobile, motor block, postoperative nausea and vomiting and sedation scores are also assessed regularly and recorded.

- During the post operative period any complications of epipleural/paravertebral infusion are noted by nursing staff. Advice from the acute pain team and the anaesthetist should be sought if pain control if problematic.

- In the event when epipleural/paravertebral infusion is deemed ineffective, morphine boluses including morphine PCA should be prescribed.
